# Application of a Multi-Gas Detector for Monitoring Gas Composition in Minced Beef During Storage

**DOI:** 10.3390/foods13223553

**Published:** 2024-11-07

**Authors:** Aleksandar Veličković, Lorenzo Cocola, Massimo Fedel, Bojana Danilović, Massimo De Marchi, Luca Poletto, Dragiša Savić

**Affiliations:** 1 Faculty of Technology, University of Niš, Bulevar Oslobodjenja 124, 16000 Leskovac, Serbia; aleksandar.velickovic@student.ni.ac.rs (A.V.); savic@tf.ni.ac.rs (D.S.); 2 Department of Agricultural and Food Studies, Toplica Academy of Applied Studies, Ćirila i Metodija 1, 18400 Prokuplje, Serbia; 3 CNR Institute for Photonics and Nanotechnologies UOS Padova, Via Trasea 7, 35131 Padova, Italy; lorenzo.cocola@cnr.it (L.C.); massimo.fedel@cnr.it (M.F.); 4 Department of Agronomy, Food, Natural Resources, Animals and Environment (DAFNAE), University of Padova, Viale dell’Università 16, 35020 Legnaro, Italy; massimo.demarchi@unipd.it

**Keywords:** multi-gas detector device, beef meat, ammonia, hydrogen sulphide, microbial count

## Abstract

This study aims to assess the capability of using a specially designed device to monitor changes in gas concentration (CO_2_, NH_3_, H_2_S, and O_2_) in the atmosphere above the minced beef meat, during storage at refrigerated temperature. With its array of sensing channels, the multi-gas detector device facilitates the detection of precise gas concentrations in sensitive environments, enabling the monitoring of various processes occurring within stored meat. To delve into the connection between microbial activity and gas emissions during storage, fluctuations in microbial populations in the meat were observed, focusing on prevalent meat microbiota such as lactic acid bacteria (LAB) and *Enterobacteriaceae*. A significant reduction of O_2_ content in the stored samples was observed after seven days (*p* < 0.05), while a significant release of CO_2_ was detected on the fourth day of storage. Significant changes (*p* < 0.05) in the gas content were tracked until the 11th day of storage followed by intensive microbial growth. NH_3_ and H_2_S levels remained undetectable throughout the experiment. The results showed a correlation between an increase in gas content in the headspace and an increase in the number of LAB and *Enterobacteriaceae* in meat. Modern multi-gas detector devices can indirectly determine microbial contamination in closed meat packaging.

## 1. Introduction

A combination of complex chemical reactions (autolytic enzymatic reactions and lipid oxidation) and biological activities occur in the meat between slaughter and usage. Due to its composition (high nutrient and moisture content, pH = 5.5–6.5), raw meat represents the perfect medium for the growth of a large range of various microorganisms [1]. Spoiled meat loses its original nutritional value, texture, or flavour, and can pose health risks to consumers. Spoilage bacteria grow in large numbers, decomposing food and altering sensory characteristics, which affects product quality. While spoilage bacteria typically do not cause illness, consuming them in high concentrations can lead to gastrointestinal discomfort. Pathogenic bacteria, on the other hand, cause infections but may not alter a food’s appearance, smell, taste, or texture, making contamination hard to detect. Foodborne illnesses from pathogenic bacteria come in two forms: food intoxication, from consuming toxins produced in the food; and food infection, from ingesting bacteria that then produce toxins in the digestive system [2].

The microbial spoilage depends on the metabolic potential of present microorganisms to produce spoilage-associated metabolites, such as fatty acids, organic acids, ketones, alcohols, sulphur compounds (hydrogen sulphide, methyl sulphide, and dimethylsulphide), ethyl esters, aldehydes, and other compounds [3,4]. So, based on a determination of some chemical parameters, it is possible to monitor the stage of meat spoilage. Additionally, the limits of such chemical markers can be estimated to facilitate monitoring of the early detection of meat spoilage and even meat quality.

Detection of the spoilage-associated molecules is not easy to predict the level of meat contamination, because interactions between molecules can yield unpredictable off-odours. Additionally, microorganisms can interact in the meat system to produce metabolites that may have a negligible role in meat spoilage [4]. Contrary to that, the realised gases are the result of microbial metabolic degradation and meat spoilage. CO_2_, NH_3_, H_2_S, and H_2_ are the result of deamination, decarboxylation, and hydrolysis of meat components, mostly proteins. The amount and ratio of gases vary greatly with the types of microorganisms and amino acids and the redox potential of the food (presence of O_2_ in atmospheres) [5].

Some studies have been conducted to elucidate the relationship between gas content in the atmosphere of food packages and the level of product quality and safety, process monitoring, and even authenticity assessment. Danilović and colleagues [6] demonstrated that CO_2_ formation during food storage is correlated with an increase in yeast populations, particularly in yogurt and other dairy products susceptible to yeast contamination. The study highlights the potential of using tunable diode laser absorption spectroscopy (TDLAS) as a simple and non-destructive method for detecting spoilage in dairy products. The results suggested a significant correlation between CO_2_ levels and yeast contamination, providing a more efficient and precise means of monitoring food quality and safety. Non-destructive measurement systems can effectively monitor O_2_ and CO_2_ concentrations in the headspace of food packages in real time, with minimal deviations from traditional destructive methods. The infrared-based systems for measuring CO_2_ showed reliability in observing variations linked to the unique microbiota on the samples. These systems are valuable for detecting premature microbial spoilage caused by contamination or cold chain interruptions. Significant changes in CO_2_ levels can be detected before sensory spoilage occurs [7]. Lovestead and Bruno [8] demonstrated that the identified compounds in the headspace of spoiled chicken can serve as indicators of spoilage, directly linking gas content in packaging to food quality. The detection of these compounds before noticeable changes in odour can facilitate early spoilage detection, which is crucial for maintaining food quality and safety. Since specific gases are only present in spoiled meat, their presence can indicate a reduction in product quality and inadequate storage conditions. The aerobic bacterial count can indicate the extent of microbial spoilage in perishable foods, with the delay in O_2_ depletion and CO_2_ accumulation in the package headspace potentially serving to estimate the shelf life and microbial stability of food [9]. The progress of the headspace gas mixture of the raw meat has been monitored mostly by using gas sensors combination (so-called ‘electronic noses’) supported by statistical analysis techniques (usually Neural Network algorithm) for meat quality assessment [10], classifying the patterns of raw beef [11], and chicken freshness detection [12,13,14]. Metal oxide semiconductors, organic conducting polymers, and piezoelectric crystal sensors are used in commercial gas sensor analysers [10]. The electronic nose offers numerous advantages for food quality control, particularly in assessing meat freshness. It allows the swift identification of spoilage or contamination, which is crucial in preventing the distribution of spoiled items. It delivers dependable results in detecting odour variations and the presence of spoilage. Research indicates a strong correlation between its findings and microbiological analyses, positioning it as an effective tool for promptly identifying spoilage without requiring lengthy laboratory processes. Moreover, the electronic nose analyses the gases above the meat, facilitating a non-invasive approach and minimizing contamination risks [15]. On the other hand, electronic nose technology presents various limitations that impact its efficacy. It is influenced by environmental factors such as temperature and humidity, which can diminish precision. It frequently encounters issues with specificity, and sensor drift may undermine dependability. Interference from other volatile compounds can obstruct analysis, and the complexity of the data necessitates sophisticated algorithms. Furthermore, some sensors possess a short operational lifespan, resulting in frequent replacements, while high expenses may hinder widespread use. Additionally, the absence of standardized procedures can lead to discrepancies in results. Although there is potential in gas analysis, these obstacles must be resolved to improve accuracy and dependability [16,17,18]. A comprehensive and thorough review of various set-ups used for gas-sensing analysis, which have been utilized in the past decade of gas-sensing measurements, can be found in the reference paper [19].

Searching for simple, selective, sensitive, inexpensive, non-destructive methods for food safety analysis has been directed at developing TDLAS. This non-invasive method measures gas content in closed containers by passing a laser beam through the headspace inside the package [6]. Practical applications in the food industry were documented for in-line monitoring of fermentation processes in beer, sparkling wine, and the soft drink industry, leak detection and closure tightness detection, and monitoring gas mixture in modified atmosphere packages [20,21,22,23,24,25]. By TDLAS determining CO_2_ content in the headspace of yogurt cups and bottles during storage, the correlation between the accumulated CO_2_ and contamination by yeasts was indicated [11]. Also, the rise of the content of ammonia in the headspace and the increase of the number of bacteria (total number of mesophilic and lactic acid bacteria) were correlated in minced beef during storage at room temperature [26].

In this paper, we present an application of a recently developed device that enables the discrimination of variations in the concentration of four gases (CO_2_, NH_3_, H_2_S, and O_2_) in the headspace of a sealed container with meat stored at 4 °C for 15 days. The device, called a multi-gas detector, is based on different sensing channels, and enables the detection of specific gas concentrations in critical environments, thereby allowing monitoring of different processes in meat stored inside. Contrary to the other devices used in the detection of gases in meat spoilage as electronic nose, or commercial gas sensor analysers, the developed multi-gas sensor is constructed to allow easy, economical, and in-line detection of realised gases without disruption of the sample. To understand the relationship between microbial presence and gases released during storage, we monitored changes in microbial populations within meat by identifying the most common meat microbiota: lactic acid bacteria and *Enterobacteriaceae*.

## 2. Materials and Methods

### 2.1. Construction of the Device

A multi-gas detector device was developed to measure the concentrations of CO_2_, NH_3_, H_2_S, and O_2_ in the atmosphere above minced beef. The constructed device aimed to utilize different sensing channels based on various principles: non-dispersive infrared spectroscopy for CO_2_ detection; a Pellistor or catalytic bead sensor for monitoring NH_3_ and H_2_S; and an electrochemical cell as an oxygen sensor.

The measurement system consists of a series of four gas sensors—each dedicated to detecting one of the following gases: oxygen, hydrogen sulphide, carbon dioxide, and ammonia. These sensors are configured in a sequential, closed-loop arrangement where the gas sample passes through each sensor in turn and eventually returns to the sample chamber.

**Figure 1:**
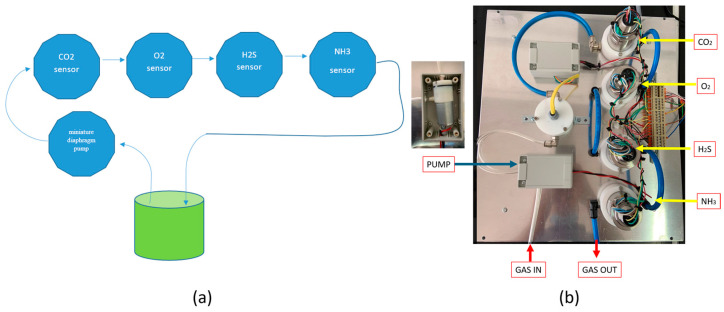
A schematic diagram (**a**), and hardware set-up (**b**) of the multi-gas loop detector system.

The gas flow within the closed loop is actively maintained by a peristaltic pump, which circulates the sample gas mixture through the sensor array. This recirculation enables a continuous dynamic equilibrium between the headspace gases within the sample chamber and those within the sensor loop. Consequently, the gas concentrations detected by the sensors consistently reflect the real-time composition of the sample’s headspace, ensuring reliable and responsive measurements. The system’s architecture is engineered to support real-time monitoring of O_2_, H_2_S, CO_2_, and NH_3_ levels without consuming or significantly altering the sample itself. By maintaining a closed and stable environment, this design minimizes potential external influences, thereby enhancing the accuracy and reproducibility of the gas measurements. A schematic diagram and hardware set-up of the multi-gas loop detector system is presented in Figure 1. This schematic diagram (Figure 1a) represents the overall layout of the multi-gas loop detector, illustrating the flow of gas from the sample jar (at the centre) through the various sensors (CO_2_, O_2_, H_2_S, and NH_3_) connected in sequence. A miniature diaphragm pump circulates the gas through the loop. The arrows indicate the flow direction. The hardware set-up of the multi-gas loop detector system is presented in Figure 1b. Figure 1b shows the actual hardware configuration of the multi-gas loop detector system. Each component in the schematic (Figure 1a) is highlighted in the image, including the CO_2_, O_2_, H_2_S, and NH_3_ sensors, the miniature diaphragm pump, wiring, and tubing connections.

**Figure 2:**
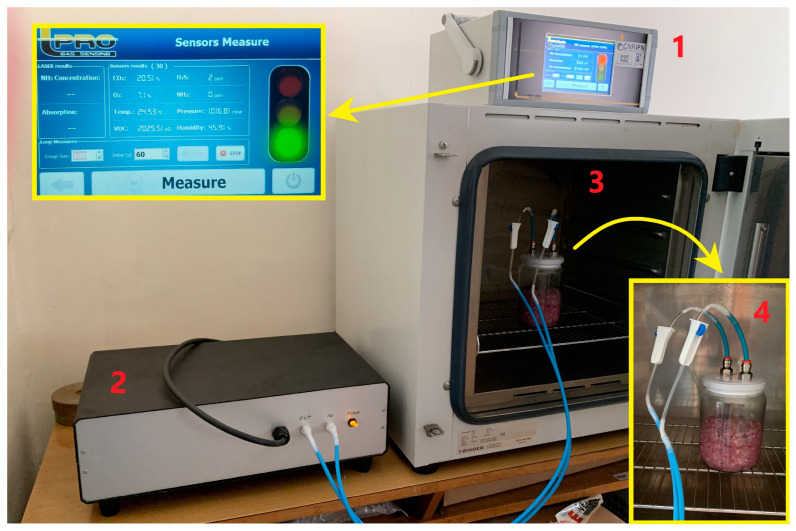
Specially designed multi-gas detector device for monitoring gas content in the headspace above minced beef meat during storage (unit for data acquisition (1); gas chamber (2); thermostat (3); and jars with minced beef (4)).

The final set-up of the system is presented in Figure 2. The device consisted of a unit for data acquisition (number 1 in Figure 2), and a closed gas chamber (number 2 in Figure 2).

### 2.2. The Meat Preparation

The proximate chemical composition of minced beef meat determined by the ISO standard methods was: 68.19% moisture [27]; 22.44% proteins [28]; and 2.61% fat [29]. The beef round was supplied from the local slaughterhouse (TP Mesokombinat-promet D.O.O. Leskovac, Serbia), 24 h post-mortem. The meat was cut into pieces (average weight between 200 and 300 g) and exposed to the action of a UV lamp (Germicidal lamp GL 15, Fatex, Vučje, Serbia), for 15 min at a distance of 6 cm from the meat sample on each side to reduce the number of the present microbiota as described by Kim et al. [30]. The parameters of the UV lamps were set as 254 nm wavelength, 150 mm length, and 15 W total power selected according to the literature data on the effectiveness of UV irradiation in meat sterilization [30,31].

After that, the meat was processed with a sterile stainless-steel meat grinder (König, Graz, Austria) in a sterile environment, which was ensured by sterilizing the grinder in an autoclave at 121 °C for 15 min. The minced meat was spread on the bottom of sterile jars to reach the same depth and surface area (number 4 in Figure 2) in all jars. A total number of 30 jars were prepared and stored under refrigeration temperature (4 °C) and gas and microbiological analyses were performed after 0, 3, 4, 7, 8, 9, 10, 11, 14, and 15 days of storage. On each sampling day, three jars were randomly selected; first, the concentration of gases was measured using a multi-gas detector device, then the jars were opened and a meat sample was taken from each jar for microbiological analysis.

### 2.3. Gas Measurement

The experiment was conducted in a multi-gas detector (Figure 2) at 4 °C in closed jars (total volume 500 mL) filled with minced beef (230 g) (number 4 in Figure 2). For the continuous measurement of the gas content, the jars were connected by the tubes with a multi-gas detector. Before each measurement, the system of the device was purged with nitrogen, an inert gas, to avoid inaccuracies in the concentration readings of O_2_. Nitrogen was chosen because it is chemically inert and does not react with the sample or interfere with the sensor readings, unlike other gases that might alter the sample composition or sensor calibration. Purging with nitrogen effectively removes residual oxygen and other gases from the system, creating a baseline that prevents contamination from previous measurements. This process ensures that each reading accurately reflects the gas composition within the jars, enhancing the precision and reproducibility of the experiment. The lid of the jars was equipped with valves for easier connecting and disconnecting jars during multiple measurements. All of the jars were kept on a thermostat (Binder GmbH, Tuttlingen, Germany) (number 3 in Figure 2), and daily three jars were connected to the instrument to measure the gas content in the headspace. After gas measuring and disconnection, the jars were excluded and opened and the meat was subjected to microbial analysis. The triplicate samples (3 jars) were prepared for all experiments.

### 2.4. Microbial Analysis

At each sampling point, meat samples (25 g) were mixed with 225 mL of saline peptone water (0.8% NaCl + 1 g/L peptone) and shaken on a multi-functional orbital shaker (PSU-20i, Biosan SIA, Riga, Latvia) for 15 min at a rotation speed of 120 rpm. The determination of the number of bacteria was performed by the standard method of serial dilution [32]. The serial dilution technique involved the preparation of a series of 10-fold dilutions, to estimate the number of bacteria in the sample expressed as colony-forming units (CFU). The volume of 1 mL of appropriate dilution was plated on De Man–Rogosa–Sharpe (MRS) agar plates (Himedia, Mumbai, India) for lactic acid bacteria, Violet Red Bile Glucose (VRBGA) agar plates (Torlak, Belgrade, Serbia) for enterobacteria, and Pseudomonas Agar Base CM0559 (Oxoid, Basingstoke, UK) with Pseudomonas CFC Selective Agar Supplement (Merck KGaA, Darmstadt, Germany) for enumeration of *Pseudomonas* spp. The supplement was added according to the manufacturer’s instructions. After the incubation of 48 h at 37 °C, the plates were enumerated. The total number of microorganisms in the sample was calculated using the formula CFU/mL = CFU × dilution factor × 1/aliquot, with an ideal count ranging from 30 to 300 colonies per plate to ensure accuracy. All experiments were performed in triplicate from each of the three jars.

### 2.5. Statistical Analysis

Statistical difference between the samples was calculated by one-way ANOVA followed by Turkey’s multiple comparison test by the software SPSS 21.0 (IBM, Armonk, NY, USA). Differences were considered significant when the *p*-value was lower than 0.05. Principal component analysis (PCA) was performed to assess the magnitude of variation between time points and their relationships with observed characteristics (physicochemical and microbiological features). For measuring the degree of linear association between the analysed variables, Pearson’s correlation coefficient was used, and a 5% significance level was applied to test their significance. PCA was conducted using XLSTAT software (XLSTAT, 2014, Addinsoft, Paris, France).

## 3. Results and Discussion

### 3.1. The Device Construction and Characteristics

The multi-gas detector was designed to detect the presence and levels of specific gas (CO_2_, NH_3_, H_2_S, and O_2_) concentrations in challenging environments with response times of just a few minutes. In summary, these sensitivity characteristics were detected through a series of calibration tests, using known concentrations of each target gas, in controlled environments.

A series of cross-correlation tests were performed to verify the response specificity of each sensor in the presence of different gas concentrations. In each test, a targeted gas concentration was introduced into the measuring jar, and the responses of all sensors were recorded across 20 repeated measurements. The tests measured responses in parts per million (ppm) for H_2_S and NH_3_, while O_2_ and CO_2_ responses were recorded as percentages. The data obtained from these tests were analysed to determine the range, repeatability, resolution, and response times for each gas sensor.

Test 1: Low H_2_S Concentration (2 ppm)

A small amount of H_2_S was injected into the measuring jar, and the system was monitored for 20 measurements. The results indicated that: the H_2_S sensor alone registered a response correlating to the introduced concentration; CO_2_ and NH_3_ sensors displayed no response, with readings consistently at zero; and the O_2_ sensor response was similarly unaffected by the H_2_S presence. This test confirmed the specificity of the H_2_S sensor at low concentrations, with no cross-correlation or interference detected from the other sensors.

Test 2: High H_2_S Concentration (70 ppm)

In the second test, a significantly higher concentration of H_2_S (70 ppm) was introduced, and measurements were repeated 20 times: the H_2_S sensor continued to respond in direct correlation with the introduced H_2_S concentration; and the CO_2_, NH_3_, and O_2_ sensors showed no response, demonstrating zero cross-correlation. These results verified that even at elevated levels of H_2_S, the H_2_S sensor’s response was isolated, with no detectable cross-sensitivity affecting the other gas sensors.

Test 3: Mixed H_2_S and CO_2_ Concentrations (50 ppm H_2_S, 25% CO_2_)

For this test, a mixture of 50 ppm H_2_S and 25% CO_2_ was injected, which resulted in the following: only the H_2_S and CO_2_ sensors responded to their respective gases; the NH_3_ sensor showed no response, registering consistently at zero, confirming no cross-sensitivity to either H_2_S or CO_2_; and the O_2_ sensor’s readings remained stable, with no indication of cross-interference. This demonstrated the isolation of the NH_3_ sensor in mixed-gas conditions, highlighting that it did not respond to the presence of H_2_S or CO_2_ in the loop.

Test 4: High NH_3_ Concentration (240 ppm)

In the final test, a high concentration of NH_3_ (240 ppm) was introduced to the measuring jar: the NH_3_ sensor registered a response directly correlated to the concentration; and the H_2_S, O_2_, and CO_2_ sensors remained unaffected, with stable readings throughout the 20 repeated measurements. This confirmed that the response of the NH_3_ sensor was independent and did not influence or interfere with the other sensors, even at elevated concentrations.

#### Summary of Cross-Correlation Results

Across all four tests, no cross-correlation or cross-sensitivity was observed among the sensors. Each sensor’s response was independent, demonstrating a high specificity for its target gas. This isolation is critical to the device’s operation, ensuring accurate, gas-specific measurements within the system.

These findings validate the device’s construction, as the absence of cross-correlation allows for reliable multi-gas monitoring without compromising individual sensor performance. The specifications of the multi-gas device are presented in Table 1.

Table 1 presents an overview of the four sensors integrated into the multi-gas detection device, each chosen based on its ability to accurately measure the target gases without cross-interference. The sensors used in this study were based on different measurement principles, tailored to detect specific gases: CO_2_, NH_3_, H_2_S, and O_2_.

CO_2_ Sensor–Non-dispersive Infrared (NDIR): For CO_2_ detection, we used an NDIR sensor model NET32-AIN-INP20-CO2T from Zeta Alarms System (Swansea, UK). This sensor utilizes a dual-wavelength infrared source and two pyroelectric detectors to precisely measure CO_2_ concentrations, with minimal cross-sensitivity to NH_3_ and H_2_S. The sensor detects CO_2_ at an absorption peak around 4.26 µm. The NDIR principle ensures high selectivity and accuracy in environments where multiple gases are present.

NH_3_ and H_2_S Sensor–Pellistor (Catalytic Bead): NH_3_ and H_2_S concentrations were measured using a NET32-ATX-NT-NH_3_-H_2_S catalytic bead (Pellistor) sensor from New Electronic Technology (N.E.T. srl., Padova, Italy). This sensor is designed to detect combustible gases, including NH_3_ and H_2_S, within a concentration range of 0–20 ppm, and is optimized for low temperature and humidity dependence. The sensor’s stability under fluctuating environmental conditions was a key factor in its selection, as it ensured reliable detection of gases produced during meat contamination.

O_2_ sensor–Electrochemical Cell: For oxygen monitoring, we employed an electrochemical cell sensor, model NET3X-AO2-O2-A2, also from N.E.T. srl. Electrochemical sensors are known for their selectivity and minimal cross-sensitivity to water vapour, making this sensor ideal for the wet, variable environment of meat storage containers. The sensor’s design is optimized for high accuracy under varying pressures and temperatures, and it is commonly used in food safety applications due to its reliability in detecting oxygen concentration changes linked to microbial activity.

Focusing on the Non-dispersive Infrared (NDIR) principle, the CO_2_ sensor employs a dual-wavelength technique using an infrared energy source directed at two pyroelectric detectors. In the mid-infrared absorption spectrum, CO_2_ has a distinct absorption band around 4.26 µm, which does not overlap with other gases in this experiment, such as NH_3_ (2.25 µm and 3.03 µm) or H_2_S (1.57 µm and 3.72 µm). This separation of absorption bands minimizes cross-sensitivity, allowing the NDIR sensor to selectively measure CO_2_ concentrations without interference from NH_3_ and H_2_S.

**Figure 3:**
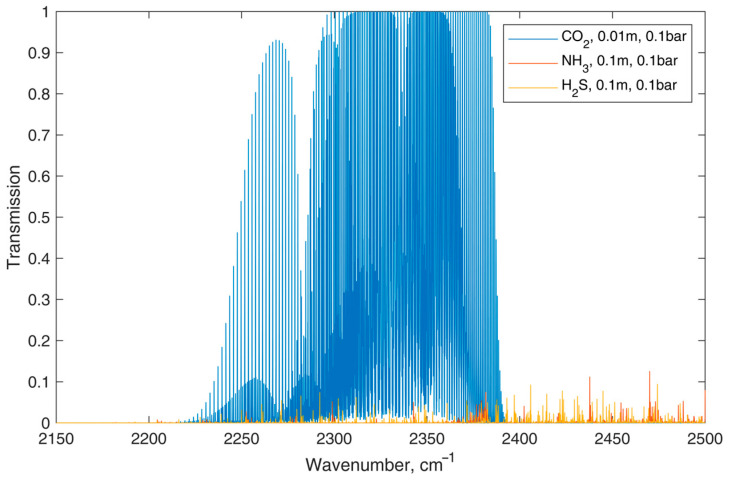
The absorption spectra of CO_2_ in comparison to NH_3_ and H_2_S. The absorption spectra of NH_3_ and H_2_S have been amplified tenfold to visually highlight the differences.

The infrared absorption spectra of CO_2_, NH_3_, and H_2_S exhibit minimal overlap, allowing these gases to be detected without cross-interference. Figure 3 illustrates the absorption spectra of CO_2_ in comparison to NH_3_ and H_2_S. The absorption cross-section of CO_2_ is significantly higher than that of the other gases, and it remains unaffected by their presence. Each gas has distinct absorption wavelengths. For instance, CO_2_ primarily absorbs in the mid-infrared region around 4.26 µm, while NH_3_ has strong absorption at 2.25 µm and 3.03 µm, and H_2_S absorbs at 1.57 µm and 3.72 µm. These absorption characteristics are well-separated, minimizing cross-sensitivity in detection systems such as Non-dispersive Infrared (NDIR) sensors [33].

During the selection process, the focus was placed on cross-sensitivity for NH_3_ and H_2_S. These gases were both expected to be present in the experimental set-up and were generated by biochemical processes during meat contamination. Pellistor sensors (N.E.T. srl. NET32-ATX-NT-NH_3_-H_2_S) with extremely low humidity and temperature dependence were selected. Pellistors or catalytic bead sensors provided an effective solution for monitoring NH_3_ and H_2_S in the range of 0–20 ppm. In the multi-gas detector, monitoring the concentration of O_2_ was also crucial. In a wet environment, the volume concentration of O_2_ (nominally 20.9% in dry air) could be affected by the temperature dependence of water vapour pressure [34]. Biochemical processes inside the meat containers also led to a reduction in O_2_ concentration. The oxygen sensors (NET, NET3X-AO2-O2-A2) installed in this multi-gas device were based on the principle of an electrochemical cell and were optimized for minimal cross-sensitivity to water vapour. Unlike partial pressure oxygen sensors, this design has good pressure and temperature dependence, making it a suitable choice for safety applications and harsh environments. Many electrochemical oxygen sensors have been reported for use in food analysis [35].

Cross-correlation tests were conducted to evaluate the performance of the multi-gas device when exposed to the different gases in the measurement loop (Table 2). When a large concentration of H_2_S was inserted into the measuring loop, no cross-correlation was observed. High concentrations of H_2_S and NH_3_ were introduced into the measurement loop separately, and no noticeable changes were observed in the concentrations detected by the other sensors. The responsivity of the other gas sensors remained stable. Tests showed that cross-correlation was not detectable at the tested working points. The multi-gas device had a low enough cross-sensitivity between H_2_S and NH_3_ to allow for simultaneous detection of different gases at ppm levels.

### 3.2. Gas Content and Microbial Composition During the Meat Storage

**Figure 4:**
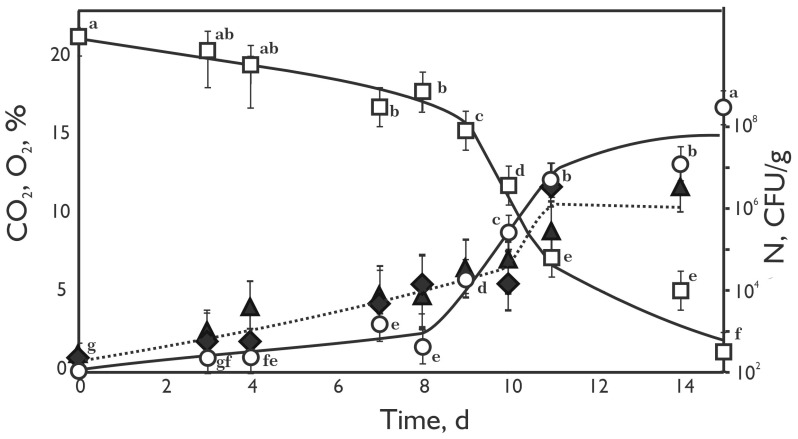
Changes of gas content (CO_2_–○; O_2_–□) in the headspace of closed jars (total volume 500 mL) and the number of bacteria (lactic acid bacteria–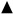; enterobacteria–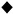) in minced beef meat (230 g) during incubation at 4 °C. The error bars represent standard errors in experimental data. Values followed by different letters (from “a” to “g”) indicate statistically significant differences at *p* < 0.05 (Tukey’s HSD test).

The changes in gas content in the headspace and microbial composition within the minced beef meat during storage are shown in Figure 4. The experiments were performed for 15 days at a temperature of 4 °C. The common temperature used in refrigerators (4 °C) was chosen to elucidate the differences influencing minced meat stability during storage.

There are numerous published data presenting microbiota normally associated with meat and meat products before spoilage [1,3,36]. Among bacteria, gram-negative bacteria are predominant, while the enterococci and lactobacilli are the gram-positive microbiota most often found. *Enterobacteriaceae* are frequently present in refrigerated meat products [37]. The LAB and enterobacteria were linearly accumulated from an initial number of 2.5 ± 0.2 × 10^2^ CFU/g to a final 7.2 ± 2.2 × 10^5^ CFU/g. Although *Pseudomonas* species can often be found in meat, the present analysis did not detect *Pseudomonas* spp. during storage. Literature data have shown that *Enterobacteriaceae* predominantly outcompete *Pseudomonas* spp. [38], which may explain the absence of *Pseudomonas* species in the analysed meat samples. Temperature and O_2_ availability are key factors influencing the selection of microbes that cause spoilage, including *Enterobacteriaceae* and lactic acid bacteria as the main bacterial groups, as shown in various studies [1,39,40].

Meat spoilage is generally correlated with the increased production of nitrogen-containing products of protein and sulphur-containing amino acids degradation, which in the end results in the increase of NH_3_ and H_2_S content [41,42]. Microorganisms that are primarily responsible for the mentioned processes mostly belong to the *Pseudomonas* spp. [43,44]. Taking into consideration that *Pseudomonas* strains were not detected in this research, notable amounts of NH_3_ and H_2_S can not be expected. Additionally, LAB is generally regarded as having low lipolytic and weakly proteolytic activity compared to species from the *Pseudomonas* genus [45]. Low temperatures reduce the enzymatic activity of LAB, so prolonged storage at low temperatures may lead to an irreversible loss of proteolytic enzyme activity [46], and ultimately cannot significantly contribute to the production of gases such as NH_3_ and H_2_S. A significant reduction of O_2_ content in stored samples was observed after seven days (*p* < 0.05), while a release of CO_2_ was detected on the fourth day of storage. The gases’ content significantly changed (*p* < 0.05) until the 11th day of storage, followed by intensive microbial growth, which can explain such high changes in headspace gas composition. The O_2_ content reached 1.5 ± 0.4%, while CO_2_ level rose to a final 16.9 ± 0.6% at the end of the monitored period (15th day) (Figure 4). The O_2_ consumption and CO_2_ generation are predominantly the result of activity of present microorganisms. In addition to microbial activity, oxygen levels can decrease in sealed packaging due to several other factors. First, oxygen binds to myoglobin (Mb) to form oxymyoglobin (MbO_2_), which is essential for maintaining the meat’s red colour. However, the oxidation of myoglobin or oxymyoglobin leads to the formation of metmyoglobin (MMb) through a process known as autoxidation, resulting in discoloration. Additionally, oxygen levels are reduced by mitochondrial respiration within muscle cells. The interaction between the diffusion of oxygen into the meat and its chemical reactions with myoglobin significantly affects oxygen consumption, ultimately impacting the meat’s colour stability and overall quality during storage [47]. Mortazavi et al. [48], showed that during the storage period, the concentrations of CO_2_ and O_2_ gases in the headspace above the meat change significantly, with CO_2_ starting to rise sharply after just three days and continuing to increase throughout 21 days of storage at 4 °C. In the same research, the O_2_ level dropped to zero after 14 days of storage, which is similar to our results. The increase in CO_2_ and decrease in O_2_ levels observed in this study align with findings by Mortazavi et al. [49], who documented rising CO_2_ and declining O_2_ levels during meat storage, which are associated with microbial metabolism. Numerous published data indicate that lactic acid bacteria are responsible for meat spoilage and that they utilize the available O_2_ and emit more CO_2_ into the packaging headspace [50,51,52]. Doulgeraki et al. [53], stated that the presence of lactic acid bacteria contributed to meat spoilage when the CO_2_ level in the packaging is elevated. Enterobacteriaceae and LAB presence was directly tied to O_2_ consumption and CO_2_ production, consistent with studies showing LAB’s fermentative capabilities and *Enterobacteriaceae*’s early-stage spoilage activity in similar environments [53]. The growth of many spoilage bacteria, including *Enterobacteriaceae*, can be reduced when there is at least 30% CO_2_ concentration in the packaging headspace [54]. The significance of Hassoun et al. [55] lies in its focus on innovative techniques for monitoring quality changes in meat and fish during traditional processing. By integrating advanced analytical methods and real-time monitoring, this study highlights the potential to enhance food safety and extend product shelf life. Ammor et al. [56] demonstrated the effectiveness of FTIR spectroscopy for rapid spoilage detection in minced beef, with findings that align with our study on how lactic acid bacteria and Enterobacteriaceae impact oxygen depletion and CO_2_ accumulation during storage. The findings from the conducted experiments indicate a relationship between the rise in gas levels in the headspace and the escalation in the count of LAB and *Enterobacteriaceae* found in minced beef meat. Consequently, employing advanced multi-gas detector devices for gas content measurement can indirectly ascertain microbial contamination in sealed meat packaging. The results align with previous studies that have explored the relationship between gas content in food packages and product quality, safety, process monitoring, and authenticity assessment, emphasising the importance of gas measurements as indicators of spoilage and overall product integrity.

### 3.3. Principal Component Analysis of the Results

**Figure 5:**
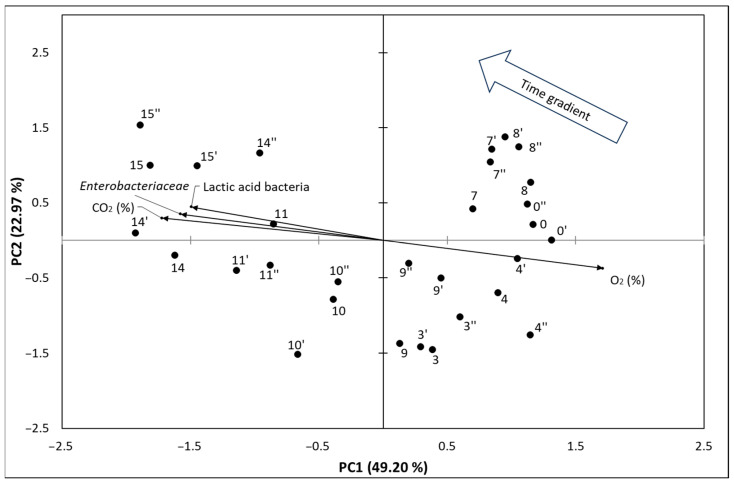
PCA biplot graph representing time points and measured parameters on 4 °C in space of the first two principal components. These two components explained 49.20% and 22.97% of the variance, respectively. The vector’s direction and length indicate the feature’s contribution to the first two components. Also, the cosine of the angle between the vectors that represent features indicates the correlation between them. Sharp angles between two vectors indicate a positive correlation with corresponding features, while obtuse angles indicate a negative correlation. The numbers (i.e., black dots) represent repetitions at specific time points.

Figure 5 depict PCA biplot graphs representing time points and measured parameters at 4 °C in the space of the first two principal components.

In Figure 5 the projection of data onto the PC1 × PC2 plane (biplot) absorbs 73.77% of the total variance, with PC1 and PC2 explaining 49.20% and 22.97% of the variance, respectively. This substantial portion of variance captured by these two principal components allows for meaningful insights into the relationships among the variables. From the figure, a strong positive correlation is observed between both types of bacteria (lactic acid bacteria and *Enterobacteriaceae*) and CO_2_ levels, while there is an inverse relationship between these bacterial populations and O_2_ levels. This suggests that as microbial activity increases, O_2_ is depleted while CO_2_ is produced, a hallmark of anaerobic microbial growth during meat spoilage.

Over the time points, there is a clear trend showing increased microbial levels, which reaches its peak on the 15th day of storage, indicating a cumulative effect of bacterial growth on gas composition. The time gradient arrow plotted on the graph provides additional insight into the temporal progression of spoilage. Feature vectors that align with the time gradient arrow (indicating the time course) signify growth dynamics, while those pointing in the opposite direction suggest periods or factors associated with declining bacterial activity or slower growth rates.

This time-point analysis has practical implications for understanding meat spoilage: for example, it demonstrates that high levels of CO_2_ and reduced O_2_ can serve as potential indicators for advanced spoilage stages. The graph also suggests that spoilage dynamics are not constant but accelerate as bacterial populations reach a critical threshold, where gas exchange and microbial activity lead to substantial biochemical changes in the meat. Monitoring these changes in real time could allow for predictive spoilage modelling and early interventions in storage conditions, particularly in commercial settings where prolonged storage can significantly impact food quality and safety.

Table 3 presents the linear correlation coefficients between the analysed parameters at 4 °C.

LAB and *Enterobacteriaceae* play crucial roles in the metabolic processes during meat storage. LAB, primarily known for their fermentative capabilities, utilize available oxygen to generate energy, although they can also function anaerobically by converting sugars into lactic acid. This metabolic flexibility allows LAB to thrive in varying oxygen conditions. Conversely, *Enterobacteriaceae* can utilize oxygen during their metabolic processes, particularly in the early stages of meat spoilage, which leads to the production of off-flavours and undesirable changes in meat quality. Their oxygen consumption can promote the growth of spoilage organisms, especially in the presence of LAB, which outcompete them when oxygen levels decrease. This dynamic underscores the importance of monitoring CO_2_ and O_2_ levels during meat storage. The significant negative correlation between CO_2_ and O_2_ levels indicates that higher CO_2_ concentrations, typically found in modified atmosphere packaging, suppress the growth of *Enterobacteriaceae* while promoting LAB growth. Ultimately, managing these microbial interactions through controlled gas levels is essential for extending the shelf life and maintaining the quality of meat products [3,57,58,59,60]. The PCA analysis illustrating bacterial and gas level correlations aligns with research by Nychas et al. [58], who used similar statistical approaches to identify spoilage trends.

## 4. Conclusions

In the present study, a newly developed multi-gas detector device was utilized to monitor changes in gas concentrations in the headspace within sealed meat containers stored at different temperatures. The results revealed distinct gas concentration patterns associated with microbial activity and meat spoilage. The microbial growth of LAB and *Enterobacteriaceae* during the storage process led to a reduction in O_2_ content and a subsequent increase in CO_2_ due to the microbial metabolism of the present bacteria. Microbial growth of LAB and *Enterobacteriaceae* during the storage process induced the reduction of O_2_ content and increase of CO_2_ realised by microbial metabolism of present bacteria. *Pseudomonas* spp. was not detected during storage, which probably induced the lack of ammonia and hydrogen sulphide in the headspace above meat. The correlation between the concentrations of CO_2_ and O_2_, as well as between these gases and bacteria, provides the foundation for future models and analyses in real conditions of food packaging and storage. These data not only quantify the relationship between changes in gas composition and microbial growth, but also indicate the potential for predictive modelling of bacterial development based on easily measurable parameters such as O_2_ and CO_2_. Although the results are expected, they provide quantitative data in the specific context of the experiment, contributing to the understanding of how these changes affect microbiological quality in controlled packaging conditions. These findings complement existing models and suggest potential applications in monitoring the condition of food in real-world settings, where even small deviations in gas concentrations may indicate potential microbial degradation. This opens up possibilities for more precise and faster methods of assessing product quality during storage.

## Figures and Tables

**Table 1:** The specifications (sensitivity characteristics) of the multi-gas device.

	Detection Gas			
Characteristic	Carbon Dioxide	Ammonia	Hydrogen Sulphide	Oxygen
Sensor type	pyroelectric	Pellistors	Pellistors	electrochemical
Detection range	0–20 vol%	0–100 ppm	0–100 ppm	0–25 vol%
Repeatability % of F.S range	±2%	±10%	±2%	±2%
Resolution	0.1%	1 ppm	0.1 ppm	0.2 vol%
Typical Response Time, s	<30	<60	<30	<10

**Table 2:** Cross-sensitivities of the H_2_S sensor and NH_3_ sensor when exposed to a large amount of different gases.

	Hydrogen Sulphide, ppm		Ammonia, ppm	
Gas	Concentration	Concentration Equivalent	Concentration	Concentration Equivalent
Hydrogen Sulphide	50	50	70	70
Carbon dioxide	4000	0	4000	0
Ammonia	70	0	70	1
Oxygen	20,900	0	20,900	0

**Table 3:** Correlation matrix of measured features at 4 °C (values above the diagonal) and corresponding *p* values (values below the diagonal).

Variables	CO_2_	O_2_	Lactic Acid Bacteria	*Enterobacteriaceae*
CO_2_	1.00	−0.99 **	0.82 **	0.89 **
O_2_	<0.001	1.00	−0.85 **	−0.91 **
Lactic acid bacteria	0.004	0.002	1.00	0.87 **
*Enterobacteriaceae*	<0.001	<0.001	0.001	1.00

** Correlation is significant at the 0.01 level.

## Data Availability

The original contributions presented in the study are included in the article, further inquiries can be directed to the corresponding authors.
